# Infraslow fluctuations of sustained attention in mood disorders

**DOI:** 10.1017/S0033291725000509

**Published:** 2025-03-12

**Authors:** Tommaso Viola, Quoc C. Vuong, Stuart Watson, Richard J. Porter, Allan H. Young, Peter Gallagher

**Affiliations:** 1 Biosciences Institute, Faculty of Medical Sciences, Newcastle University, Newcastle upon Tyne, UK; 2 School of Psychology, Faculty of Medical Sciences, Newcastle University, Newcastle upon Tyne, UK; 3 Translational and Clinical Research Institute, Faculty of Medical Sciences, Newcastle University, Newcastle upon Tyne, UK; 4 Northern Centre for Mood Disorders, Newcastle University, Newcastle upon Tyne, UK; 5 Cumbria, Northumberland, Tyne and Wear NHS Trust, Newcastle upon Tyne, UK; 6 Department of Psychological Medicine, University of Otago, Christchurch, New Zealand; 7 Te Whatu Ora, Specialist Mental Health Services, Christchurch, New Zealand; 8 Department of Psychological Medicine, Institute of Psychiatry, Psychology and Neuroscience, King’s College London, London, UK

**Keywords:** mood disorders, depression, bipolar, attention

## Abstract

**Background:**

Sustained attention is integral to goal-directed tasks in everyday life. It is a demanding and effortful process prone to failure. Deficits are particularly prevalent in mood disorders. However, conventional methods of assessment, rooted in overall measures of performance, neglect the nuanced temporal dimensions inherent in sustained attention, necessitating alternative analytical approaches.

**Methods:**

This study investigated sustained attention deficits and temporal patterns of attentional fluctuation in a large clinical cohort of patients with bipolar depression (BPd, *n* = 33), bipolar euthymia (BPe, *n* = 84), major depression (MDd, *n* = 38) and controls (HC, *n* = 138) using a continuous performance task (CPT). Longitudinal and spectral analyses were employed to examine trial-level reaction time (RT) data.

**Results:**

Longitudinal analysis revealed a significant worsening of performance over time (vigilance decrement) in BPd, whilst spectral analysis unveiled attentional fluctuations concentrated in the frequency range of 0.077 Hz (1/12.90 s)–0.049 Hz (1/20.24 s), with BPd and MDd demonstrating greater spectral power compared to BPe and controls.

**Conclusions:**

Although speculative, the increased variability in this frequency range may have an association with the dysfunctional activity of the Default Mode Network, which has been shown to oscillate at a similar timescale. These findings underscore the importance of considering the temporal dimensions of sustained attention and show the potential of spectral analysis of RT in future clinical research.

## Introduction

Sustained attention is the capacity to maintain a focused state of mind whilst avoiding distractions over time. It is resource-demanding thus there is an increase in response errors, reaction time (RT) fluctuations, or slowing with increasing cognitive load or time-on-task (Warm et al., 2008). Clinical populations show a higher tendency for sustained attention failures than the general population. For example, although characterized by a broad profile of cognitive dysfunction, people with mood disorders consistently show evidence of sustained attention deficits; both in major depressive disorder and bipolar disorder relative to healthy controls (Ancín et al., 2010; Clark et al., 2002; Gallagher, 2020; Harmell et al., 2014; Paelecke-Habermann et al., 2005; Van Der Meere et al., 2007). This has been attributed to the disruption of white matter integrity that has been observed in mood disorder populations (Poletti et al., 2015; Tamnes et al., 2012). However, sustained attention is a complex, multifaceted construct (Fortenbaugh et al., 2017) and the pattern and magnitude of observed deficits may be dependent upon the demands of the task and the method for quantifying performance.

Continuous performance tasks (CPT) are widely used in clinical settings to measure sustained attention. Deficits in CPT performance have been found in symptomatic states (Fleck et al., 2012; Koetsier et al., 2002; Little et al., 2024; Porter et al., 2003), as well as in euthymia (Doyle et al., 2005; Kolur et al., 2006; Liu et al., 2002). The principal variables of interest in CPTs are typically overall performance accuracy (e.g. d-prime, or hit-rate) and mean RT. However, these measures fail to account for the intrinsic variability stemming from fluctuations in performance over time. To retain this information, it is possible to use indices such as the standard deviation of trial-by-trial RT, or the coefficient of variation (CoV) of RT which corresponds to the mean-normalized standard deviation. Few studies have assessed intraindividual variability in mood disorder, but existing evidence suggests higher variability in bipolar disorder (Kim et al., 2015) and in major depression (Naim-Feil et al., 2015; Schmidt et al., 2021) compared to healthy controls.

Distributional analysis is an alternative to this approach. It entails fitting individual-level RT data to a non-normal distribution (Costa et al., 2019) such as the ex-Gaussian. This distribution is the convolution of a Gaussian and an Exponential distribution and is characterized by three parameters: mu and sigma, respectively the mean and standard deviation of the Gaussian, and tau, the scale of the Exponential. The ex-Gaussian distribution has been shown to fit empirical RT data well (Luce, 1986) and to reveal effects which might be masked by ‘standard’ methods of analysis (Parris et al., 2013). Previously, for instance, we reported evidence of deficits in different subcomponents of sustained attention in mood disorders by fitting RT data to an ex-Gaussian distribution to estimate the three parameters (Gallagher et al., 2015), obtained from the Vigil CPT (Cegalis & Bowlin, 1991). Specifically, relative to healthy controls, the euthymic bipolar group was characterised by a significantly larger tau parameter, that is the tendency for disproportionately slow responses. The depressed bipolar group showed significantly larger sigma and tau, suggesting higher trial-to-trial variability, while the medication-free major depression group did not differ in terms of ex-Gaussian parameters. However, the functional relevance and psychological interpretation of the parameters of the ex-Gaussian distribution remains unclear (Fitousi, 2020; Heathcote et al., 1991). Crucially, such methods fail to characterize the temporal dimension of performance (Machida et al., 2022).

The vigilance decrement refers to the phenomenon of worsening performance over time (Parasuraman & Davies, 1977) which is usually attributed to either a lack of cognitive resources (Warm et al., 2008) or inadequate allocation of the available resources (Thomson et al., 2015). The vigilance decrement can be examined using several approaches; the most common method entails modeling general linear trends, by examining the variables extracted in several trial blocks over time. However, attention (VanRullen, 2018) and cognition in general (Shalev et al., 2019) work rhythmically, therefore performance variability should also be examined in terms of cyclical or oscillatory patterns of performance.

**Figure 1.**
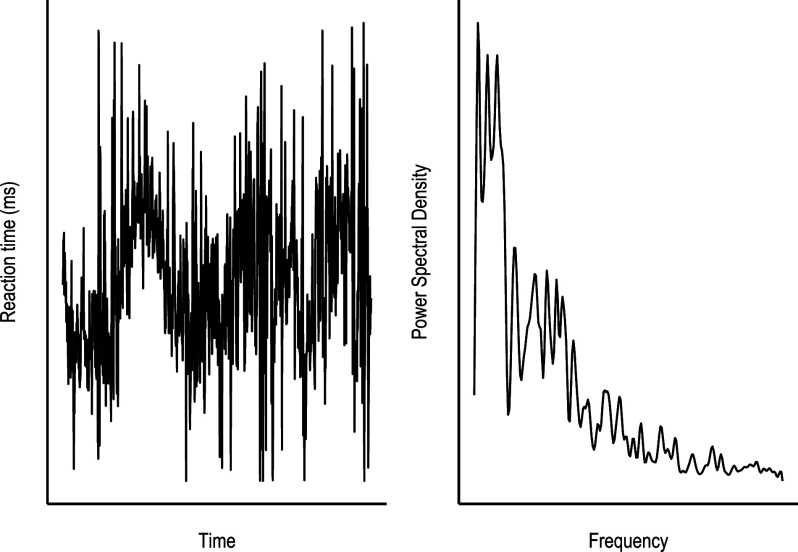
Spectral analysis transforms RT data from the time domain (A, seconds or trials) to the frequency domain (B), potentially revealing oscillatory behavior.

*Infraslow* oscillations (within a frequency range of 0.01 Hz–0.10 Hz, (Monto et al., 2008)) can be detected in both brain activity (Fox et al., 2005) and in behavioral data (Di Martino et al., 2008). In the latter, they can be obtained using the Fast Fourier Transform (FFT, Figure 1). This technique enables the extraction of the relevance (power) of oscillatory components present in the signal, each characterized by a distinct period or frequency. In the obtained curve, if any periodic recurring changes are present in RT they will be shown as peaks of power at the specific frequency. The integrated area under the power curve equates to the total variance of the RT signal. Therefore, the technique provides an examination of specific time scales within the total variance. In other words, it is possible to highlight whether performance variation follows specific temporal frequencies. Despite the relevance of this method, few studies have assessed these frequency oscillations in clinical cohorts. Frequency decomposition using FFT or wavelet analyses has been examined in attention-deficit/hyperactivity disorder (ADHD) (Karalunas et al., 2014). Compared to healthy controls, increased power is found between the interval of 0.07 Hz–0.02 Hz and centered around 0.05 Hz (Castellanos et al., 2005; Di Martino et al., 2008; Johnson et al., 2007; Vaurio et al., 2009). In other words, the increased performance variability due to attentional fluctuations found in ADHD is consistently located within a frequency range (every 14–50 s). Therefore, by utilizing spectral analysis, it is possible to develop the observation of attentional fluctuation to characterize their specific periodicity. This intraindividual variability in attention may constitute a transdiagnostic endo-phenotype (Karalunas et al., 2014). The aim of this study is to characterize in detail the vigilance decrement and periodicity of attentional fluctuation across mood disorders; specifically bipolar depression, euthymia, and major depression. Building from the distributional analysis of RT data shown previously (Gallagher et al., 2015), the novelty of our current approach is first in retaining the temporal dimension. Given the increased variability found in the clinical samples, we hypothesize a more pronounced vigilance decrement in mood disorder groups compared to healthy controls. Secondly, in order to utilize RT data from common attentional CPTs (where responses do not occur at fixed intervals) we apply a novel method of spectral analysis to characterize the periodicity of attentional fluctuations.

## Methods

### Participants

This study utilized a pooled multi-study dataset which was compiled for (Gallagher et al., 2015). We utilized this dataset as all index studies used the same attentional task, followed the same administration procedure, and were conducted by the Faculty of Medical Sciences, Newcastle University (Gallagher et al., 2014; Macritchie et al., 2010; Porter et al., 2003; Thompson et al., 2005). The dataset included a total of 296 participants between 18 and 65 years old. After the pre-processing which entailed a minimum accuracy on the attention task of 60% (see the section below for more details), the total number of participants was 293. Table 1 presents the demographics of the four diagnosis groups.

**Table 1.** Participant demographics

Diagnosis	Code	N	Mean age in years (SD)	N males
Control	HC	138	40.5 (12.50)	61
Bipolar depression	BPd	33	47.0 (8.64)	19
Bipolar euthymia	BPe	84	43.7 (9.47)	40
Major depressive disorder	MDd	38	31.6 (9.97)	14

For the bipolar cohorts, the diagnosis of bipolar disorder was confirmed using the Structured Clinical Interview for DSM-IV (SCID). Recruitment was from secondary and tertiary care services. All were out-patients and either currently in a SCID-defined depressive episode (BPd); or euthymic, prospectively confirmed at initial assessment and after 4 weeks (BPe). All were receiving medication at the time of testing, stable for 4 weeks or more. Exclusion criteria were: any other current Axis I disorder (except anxiety) or substance dependence/abuse.

For the MDD cohort, all had a DSM-IV diagnosis of major depressive disorder and were in a current depressive episode (MDd). All were recruited from general practice clinics and were psychotropic medication-free for at least 6 weeks before recruitment. Neuropsychological testing occurred as soon as possible after recruitment to minimize delay in treatment.

Healthy controls were recruited by advertisement and were demographically matched to the clinical groups. Exclusion criteria were: a personal or first-degree history of psychiatric illness, a significant untreated medical condition likely to affect cognitive function, and a current/history of alcohol/drug abuse or dependence. Fuller diagnostic and clinical details can be found in the original papers.

For all participants, illness characteristics, clinical ratings, and medication history were determined by trained psychiatrists using full history, case notes, medication review, and standardized rating scales. All studies were approved by the local National Health Service (NHS) Research Ethics Committee and all participants gave written informed consent.

### Materials and task

Each participant completed the Vigil CPT (Cegalis & Bowlin, 1991), a computerized task used to assess sustained attention in participants. In each of the 480 trials, a random letter was presented for 85 ms, followed by 900 ms of interstimulus interval (shown as a centered white letter on a dark screen). Participants were instructed to respond only to the target sequence of an “A” followed by a “K,” as quickly and as accurately as possible by pressing the spacebar. The stimulus presentation was not interrupted should the participant fail to provide a response within the response window. The total number of targets was 100, semi-randomly distributed across the length of the task (25, in each of the 4 blocks of 120 trials).

### Data analysis

All data extraction, preprocessing, and analysis were performed using the R programming language (R Core Team, 2021), in addition to multiple packages including tidyverse (Wickham et al., 2019), retimes (Massidda, 2013), lme4 (Bates et al., 2015), psych (Revelle, 2022), and lomb (Ruf & Astropy, 2022).

### Preprocessing

The preprocessing of data followed a different algorithm from that applied previously (Gallagher et al., 2015). Traditionally, RT in the Vigil CPT can have a range of 0 ms to 985 ms (the interval between the onsets of two consecutive stimuli). In this updated procedure, the window of response was increased to 1135 ms (985+ 150 ms within the following stimulus response window). The increased response window was implemented to obtain a wider range of RT as it allowed potential attentional lapses (very slow RT) that occurred after the onset of the following stimulus. The categorization of responses followed a similar procedure to a traditional Signal Detection Theory approach (Stanislaw & Todorov, 1999), which resulted in identifying correct responses (a response after the target sequence), commission errors (a response following a non-target trial), correct rejection (no responses to a non-target trial), and misses (no responses to a target trial). The procedure to obtain RT was implemented only on correct responses. Once the number of responses by a participant was obtained, an accuracy of at least 60% (60 correct responses over 100 potential target trials) was used to filter performance. This threshold was implemented because the spectral analysis requires as much available data as possible to produce stable output.

### Vigilance decrement in variability

The first step of the analysis entailed the extraction of an index of RT variability, which corresponded to the CoV (the standard deviation of RT divided by the mean RT). This was calculated for each individual participant separately using RT obtained from correct responses, in 8 blocks of 60 trials. This was preferred over utilizing 4 blocks as the change of performance over time was modeled linearly, a higher number of measures over time allow for a more reliable estimation of the trend. The CoV RT was then modeled using a mixed effect model that included the effects of grouping (Diagnosis) and time-on-task (Block, mean-centered to allow a more meaningful interpretation of the model terms), and their interaction. Additionally, the model included the covariates of age and sex, to control for differences between the cohorts. Finally, the model comprised a random slope and intercept for each individual to allow individual differences: the random intercept allowed each individual to have a different average CoV RT whereas the random slope allowed each participant to have a different effect of Block. The statistical significance of diagnosis and block was assessed with an F-test utilizing an approximation of degrees of freedom following the Satterthwaite’s method. If any of these factors was significant, multiple comparisons were implemented with Tukey adjustments when required. Contrasts were dummy coded with the HC group as a reference for comparisons. Measures of estimates in the model will be provided in the original scales of the variable and standardized in an ad hoc table. An approximation of model fit will be provided as marginal and conditional R^2^ (Rights and Sterba 2019).

### Spectral analysis of RT

To investigate whether differences in RT variance between the groups were located at specific time periods, group-averaged periodograms were examined. These were derived from the responses of each individual, where RT for correct responses was extracted and detrended. Due to the task requirements of the Vigil CPT, traditional frequency decomposition based on the Fast Fourier Transform is not usually straightforward to implement (although see [Gazzellini et al., 2017]). Due to the low target rate of the Vigil CPT, the ‘sampling’ of RT is uneven, making it unsuited for FFT. Instead, the Lomb-Scargle method (Ruf, 1999) was implemented in R. This algorithm is suited for unevenly sampled data and returns the power at specific frequencies, similar to that derived with an FFT. This method utilizes an automated heuristic that extracts an ideal set of frequencies, depending on the input data (range, data recordings over time).

A functional data analysis approach was used to detect any differences between groups (Ramsay & Silverman, 2005). First, the power curve of each participant obtained from the Lomb-Scargle periodogram was smoothed using a cubic spline with 46 basis (and 44 knots) (Figure 3A). This value was selected to reasonably maintain potential peaks in the curves. The functions were then evaluated at the appropriate range of frequencies (corresponding to maximum and minimum frequencies obtained from the frequency decomposition). We first conducted a functional ANOVA to determine if there were any frequencies during which the power differed between Diagnosis groups (Figure 3B). Following the functional ANOVA, post-hoc functional t-tests were run between group pairs. Two sets of critical t-values were calculated for each comparison, one uncorrected and one Bonferroni corrected. Calculations of ANOVA (F-test) and t-tests were performed from regression models that included age and sex as covariates, consistently with the analysis of the vigilance decrement in CoV.

## Results

### Vigilance decrement

**Figure 2.**
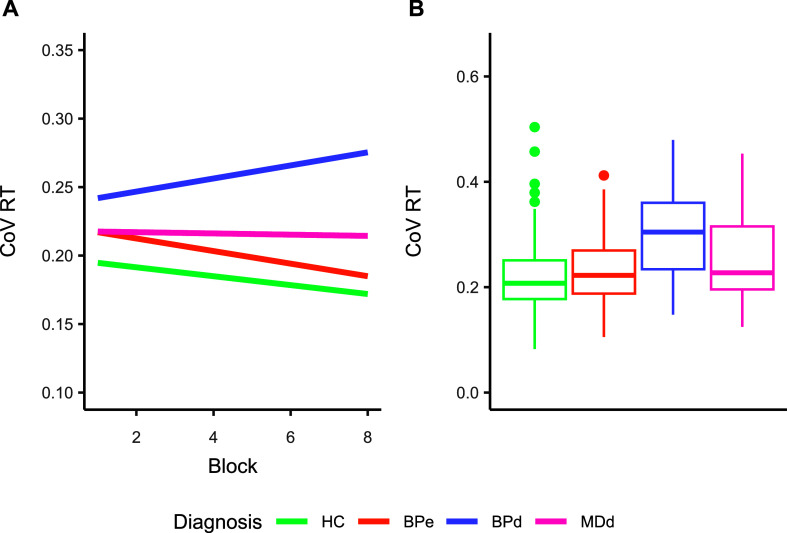
Differences in coefficient of variation (CoV) between groups. (a) CoV of reaction times modeled for the 4 groups and across blocks of trials. Dots represent the individual participants, whilst the lines are the model predictions. (b) Coefficient of Variation by group.

The mixed effect model [Marginal R^2^ = 0.06, Conditional R^2^ = 0.31] (Figure 2 and Tables 2 and 3) showed that diagnosis (F[3, 287] = 14.95, *p* < 0.001), and the interaction between diagnosis and block (F[3, 289] = 4.32, *p* = 0.005) had a significant effect on RT variability. The main effect of block (F[1, 289] = 0.90, *p* = 0.34) was not significant.

**Table 2.** Standardised parameters of the mixed effect model

Parameter	Std_Coefficient	Lower 95% CI	Upper 95% CI
(Intercept)	−0.173	−0.270	−0.076
Diagnosis [Bpe]	0.172	0.014	0.330
Diagnosis [BPd]	0.733	0.508	0.957
Diagnosis [MDd]	0.317	0.104	0.531
Block	−0.072	−0.122	−0.022
Age	−0.030	−0.101	0.040
Sex [Male]	0.004	−0.063	0.071
Diagnosis [Bpe] x Block	−0.030	−0.111	0.051
Diagnosis [BPd] x Block	0.179	0.065	0.293
Diagnosis [MDd] x Block	0.062	−0.045	0.170

**Table 3.** Summary statistics of CoV RT across blocks for the 4 groups of participants, mean (SD)

		Block							
		1	2	3	4	5	6	7	8
Diagnosis	HC	0.21	0.18	0.19	0.18	0.17	0.17	0.18	0.17
		(0.10)	(0.09)	(0.10)	(0.11)	(0.09)	(0.09)	(0.09)	(0.08)
	BPd	0.24	0.22	0.29	0.24	0.26	0.27	0.26	0.27
		(0.10)	(0.13)	(0.12)	(0.12)	(0.11)	(0.15)	(0.11)	(0.13)
	BPe	0.24	0.20	0.19	0.18	0.18	0.20	0.19	0.19
		(0.12)	(0.10)	(0.08)	(0.08)	(0.10)	(0.09)	(0.09)	(0.08)
	MDd	0.25	0.21	0.20	0.21	0.21	0.20	0.21	0.24
		(0.14)	(0.11)	(0.08)	(0.09)	(0.10)	(0.10)	(0.12)	(0.12)

Pairwise comparisons of diagnosis showed that HC had significantly lower RT variability compared to BPd (estimate = −0.075, *p* < 0.001) and MDd (estimate = −0.033, *p* = 0.02), whereas it did not differ from BPe (estimate = −0.018, *p* = 0.146). BPd had significantly higher RT variability compared to BPe (estimate = −0.058, *p* < 0.001) and MDd (estimate = 0.043, *p* = 0.024). Bpe did not differ compared to MDd (estimate = −0.015, *p* = 0.61).

Post-hoc tests of the slopes revealed that HC (b = −0.0033, *p* = 0.005) exhibited a significant decrease in RT variability with time-on-task, similar to BPe (b = −0.0046, *p* = 0.002), whereas BPd showed a significant increase in RT variability (b = 0.0048, *p* = 0.042). MDd showed no significant change in RT variability over time (b = −0.00045, *p* = 0.836).

Further comparisons of the slopes across groups demonstrated that the slope for BPd was significantly different from both HC (∆b = −0.0080, *p* = 0.012) and BPe (∆b = −0.0094, *p* = 0.004). However, differences in slopes between HC and BPe (∆b = 0.0013, *p* = 0.889), HC and MDd (∆b = −0.0028, *p* = 0.668), and BPe and MDd (∆b = −0.0041, *p* = 0.396) were not significant.

### Frequency analysis of RT

**Figure 3.**
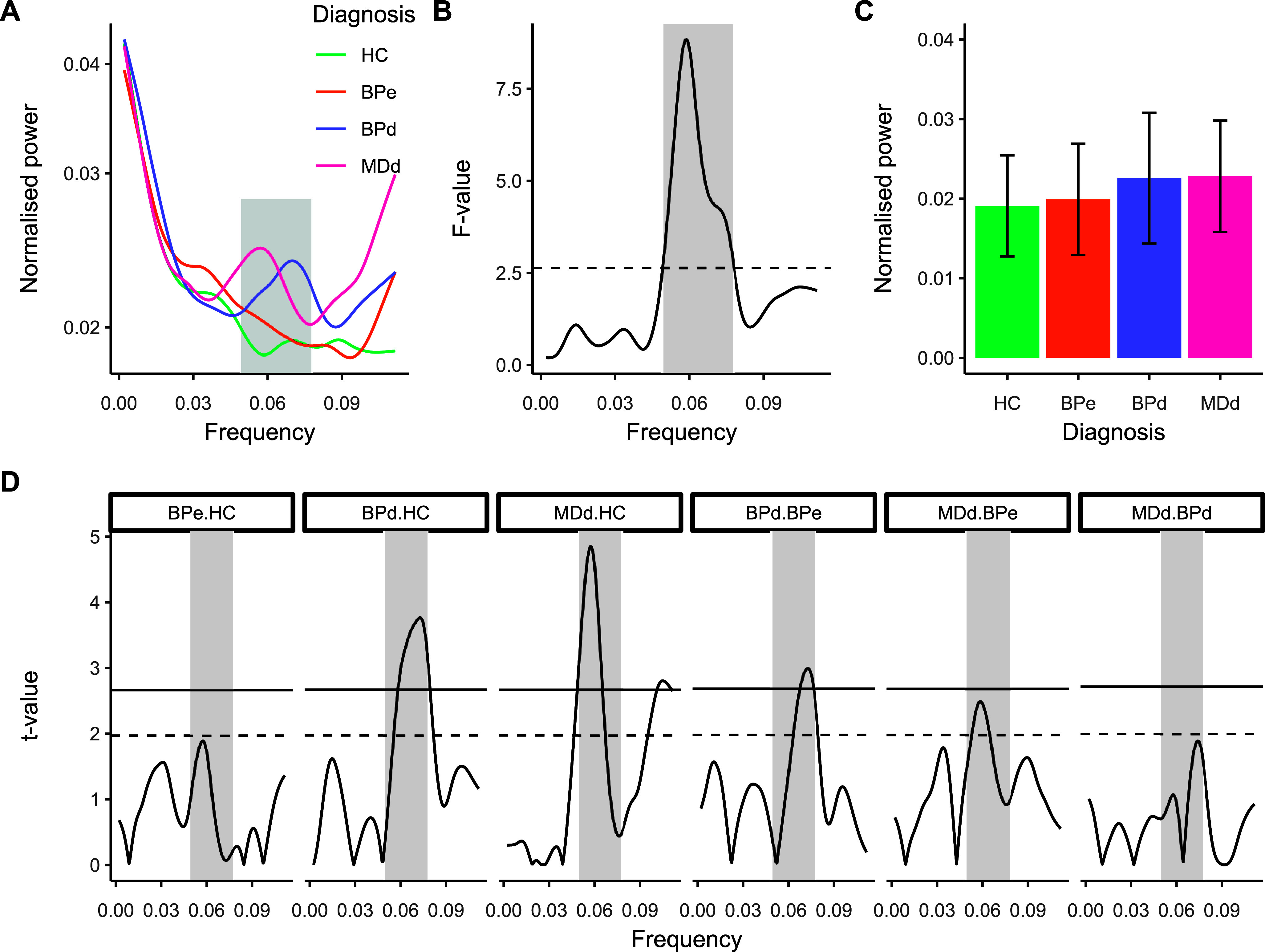
Frequency analysis of reaction time data and investigation of differences between the clinical groups. (a) averaged power curves for each experimental group. (b) functional F-test between the power curves; the dotted line represents the critical F-value; the grayed area is where the F-test is considered significant. (c) average absolute power for each group within the frequency range where the F-test was significant. (d) functional t-tests following the significant F-test; the dotted horizontal line represents uncorrected critical t-value whilst the solid line is the Bonferroni corrected value.

The functional ANOVA revealed a significant effect of diagnosis on the RT power spectrum (critical F[4, 289] = 2.64, *p* < 0.05; dashed line in Figure 3B) between 0.077 and 0.049 Hz (see gray-shaded region in Figure 3B). In the time domain, this frequency range reflects RT oscillations (e.g. lapses in attention) once every 12.90s to once every 20.24 s. Post-hoc comparisons between the groups (Figure 3C and D) within the frequency limits showed that both BPd (critical t[169] = 2.67, *p* < 0.05) and MDd groups (critical t[171] = 2.67, *p* < 0.05) were characterized by significantly greater spectral power in the oscillations than the HC group. The BPe group exhibited lesser spectral power when compared to BPd (critical t[115] = 2.68, *p* < 0.05) as well as when compared to MDd, but only using the uncorrected critical t-value (critical t[120] = 1.98, *p* < 0.05).

## Discussion

The aim of this study was to further our understanding of the nature of intraindividual variability in sustained attention in mood disorder, by measuring how response-time variability changes over time and characterizing the pattern of oscillatory behavior of RT from a continuous performance test in depressed and euthymic bipolar disorder, and major depression. Our results showed differences in sustained attention both in terms of overall variability and in vigilance decrement between the clinical samples. RT variability was highest in BPd, significantly greater than HC, BPe, and MDd. Notably, these differences were further amplified by distinct patterns of vigilance decrement: with time-on-task, variability increased in BPd, while in the other groups it either remained stable (MDd) or decreased (BPe and HC). These potential state-related effects were corroborated by the significant differences in slope between the BPd group and BPe and controls. The results of the spectral analysis complemented these findings, with a significant difference within the frequency range of 0.049 Hz (1/20.24 s) to 0.077 Hz (1/12.90 s). Within this infraslow frequency range, the BPd and MDd groups had larger power than the HC; and the BPd and MDd groups had larger power than the BPe group. However, some caution is needed when considering the MDd and BPe comparison as the differences were only significant at the uncorrected critical t-value.

The present results replicated and expanded on our previous findings (Gallagher et al., 2015), where we found that among the different samples, the depressed BP patients showed higher overall variability. Here, the analysis identified a significant main effect of diagnosis and a significant interaction with block, indicating not only robust cross-methodological differences in overall variability, but different patterns of performance over time. The inclusion of time-on-task effects is an approach that has also been recommended in ADHD research (Machida et al., 2022) as it provides a more complete profile of sustained attention performance (Esterman et al., 2014). The literature on the vigilance decrement in mood disorders is limited, particularly in terms of assessing changes in variability. Some studies have reported stable attentional performance over time in BPe (Clark et al., 2002; Fleck et al., 2001), while others have reported fluctuating or increasing differences in BPe compared to controls at later trial blocks in CPT tasks (Marotta et al., 2015; Robinson et al., 2013). This might be due to the different variables examined and the method in which performance over time was evaluated. The literature in the area displays significant heterogeneity in the implemented tasks, which are often more complex and likely to maintain a higher level of participant engagement, which is not ideal to investigate lapses in sustained attention. To our knowledge, this is the first study to examine this vigilance decrement in a sample of symptomatic bipolar disorder patients. In contrast with BPe, BPd variability increased over time. In contrast with controls and BPe, which showed potential practice effects by less variable performance, the MDd group remained stable and consistently more variable than the controls, similar to earlier studies (Van Der Meere et al., 2007). These results challenge the view of a trait-level sustained attention deficit in bipolar disorder and highlight that the deficit might be moderated by the mood state (Clark & Goodwin, 2004). Although speculative, this partially supports the notion that sustained attention depends on ‘energetic’ states such as high motivation (Warm et al., 2008), which may be compromised by depressive symptoms (Grahek et al., 2019; Smith, 2013).

Our results on the spectral analysis of RT are in line with the idea of state-related deficits of sustained attention, as symptomatic groups (BPd and MDd) showed higher variability than both controls and euthymia. More specifically, the group effect was found within the frequency range between 0.049 Hz and 0.077 Hz and (1/20.24–1/12.90 s) and centered around 0.063 HZ (15.77 s). The frequency range in which differences were observed overlapped with the range found in ADHD using similar tasks (Adamo et al., 2014; Di Martino et al., 2008; Geurts et al., 2008; Johnson et al., 2007). The range we found also overlapped with the range found for individuals who suffered traumatic brain injury (Gazzellini et al., 2017) and those who are ‘high worriers’ (Gazzellini et al., 2016).

There are important potential biological underpinnings of this frequency range across these different studies and samples. This pattern of variability has been associated with the Default Mode Network (DMN), a large-scale brain network, which comprises areas of the ventral medial prefrontal cortex, the dorsal medial prefrontal cortex, and the posterior cingulate cortex, particularly activated during introspective states (Raichle et al., 2001), and seemingly activated following the same multi-second temporal frequency (<0.1 Hz) (Fox et al., 2005). In goal-directed behavior the DMN deactivates, and the brain switches to an extrospective state where other areas (referred to as task-positive areas) are activated (Fransson, 2005). Dysfunctional behavioral oscillations in clinical conditions have therefore been explained in terms of periodic intrusion of the DMN, usually active during resting state and believed to be incompatible with effective sustained attention performance during goal-directed action (Hultsch et al., 2002). Remarkably, the DMN has been shown to fluctuate following the same time signature as behavioral lapses in ADHD (Sonuga-Barke & Castellanos, 2007; Zhang et al., 2022). Although preliminary, our results may suggest that the same dysfunctional mechanism of DMN regulation might be present in mood disorders, especially in symptomatic states. Dysregulation of the DMN has been highlighted both in major depression (Marchetti et al., 2012), and in bipolar disorder (Rodríguez-Cano et al., 2017) and our results are in line with the notion of mood state-related differences in DMN activity in bipolar disorder (Martino et al., 2016).

There are important methodological considerations to note regarding the current findings. Previous studies have used common methods such as the Fourier Transform and the Wavelet Transform to decompose oscillatory signals into power and frequencies, which could not be applied due to the CPT task characteristics. The Lomb-Scargle periodogram method used here is promising and applicable to tasks with non-regular responses (Ruf, 1999). However, few previous applications of this method exist in neuropsychological research and therefore results should be interpreted carefully. Additionally, the range of frequencies obtained from the spectral decomposition is dependent on the density of the behavioral recording. Since the target rate of the Vigil CPT task is low, the recording was sparse. Future investigations should implement tasks with higher target rate to obtain richer data in terms of frequency range and to output results that could be more easily compared to existing findings, which have settled on a specific class of CPT (e.g. the SART [Johnson et al., 2007]) (Geurts et al., 2008). Finally, future studies should consider the recording of neurophysiological measurements (e.g. EEG (Helps et al., 2008)) alongside behavioral data, as this would provide an analytical approach on the same time scale and permit a more direct assessment of the association between the brain and behavior (Gazzellini et al., 2017).

This study utilized novel methods of analysis to develop our understanding of attentional dysregulation in mood disorders. Previously, the utility of distributional modeling has been demonstrated to separate components of the RT distribution more precisely between mood disorders and healthy controls. Here, we have found important differences when temporal characteristics are retained, particularly in relation to depressive episodes and bipolar disorder. This study underscores the potential value of such approaches in characterizing sustained attention dysregulation, which could prove useful in future clinical and non-clinical research. The study also highlights that this deficit is seen in a functionally relevant metric, which may suggest a close coupling with brain network activity and represents an important direction for further research.

## References

[r1] Adamo, N., Di Martino, A., Esu, L., Petkova, E., Johnson, K., Kelly, S., Castellanos, F. X., & Zuddas, A. (2014). Increased response-time variability across different cognitive tasks in children with ADHD. Journal of Attention Disorders, 18(5), 434–446. 10.1177/1087054712439419 22508759

[r2] Ancín, I., Santos, J. L., Teijeira, C., Sánchez-Morla, E. M., Bescós, M. J., Argudo, I., Torrijos, S., Vázquez-Álvarez, B., De La Vega, I., López-Ibor, J. J., Barabash, A., & Cabranes-Díaz, J. A. (2010). Sustained attention as a potential endophenotype for bipolar disorder. Acta Psychiatrica Scandinavica, 122(3), 235–245. 10.1111/j.1600-0447.2009.01532.x 20105148

[r3] Bates, D., Mächler, M., Bolker, B. M., & Walker, S. C. (2015). Fitting linear mixed-effects models using lme4. Journal of Statistical Software, 67(1). 10.18637/jss.v067.i01

[r4] Castellanos, F. X., Sonuga-Barke, E. J. S., Scheres, A., Di Martino, A., Hyde, C., & Walters, J. R. (2005). Varieties of attention-deficit/hyperactivity disorder-related intra-individual variability. Biological Psychiatry, 57(11), 1416–1423. 10.1016/j.biopsych.2004.12.005 15950016 PMC1236991

[r5] Cegalis, J., & Bowlin, J. (1991). Vigil: Software for the assessment of attention. Nashua, NH*:* Forthought.

[r6] Clark, L., & Goodwin, G. M. (2004). State- and trait-related deficits in sustained attention in bipolar disorder. European Archives of Psychiatry and Clinical Neuroscience, 254(2), 61–68. 10.1007/s00406-004-0460-y 15146334

[r7] Clark, L., Iversen, S. D., & Goodwin, G. M. (2002). Sustained attention deficit in bipolar disorder. The British Journal of Psychiatry, 180(4), 313–319. 10.1192/bjp.180.4.313 11925353

[r8] Costa, A. S., Dogan, I., Schulz, J. B., & Reetz, K. (2019). Going beyond the mean: Intraindividual variability of cognitive performance in prodromal and early neurodegenerative disorders. Clinical Neuropsychologist, 33(2), 369–389. 10.1080/13854046.2018.1533587 30663511

[r9] Di Martino, A., Ghaffari, M., Curchack, J., Reiss, P., Hyde, C., Vannucci, M., Petkova, E., Klein, D. F., & Castellanos, F. X. (2008). Decomposing intra-subject variability in children with attention-deficit/hyperactivity disorder. Biological Psychiatry, 64(7), 607–614. 10.1016/j.biopsych.2008.03.008 18423424 PMC2707839

[r10] Doyle, A. E., Wilens, T. E., Kwon, A., Seidman, L. J., Faraone, S. V., Fried, R., Swezey, A., Snyder, L., & Biederman, J. (2005). Neuropsychological functioning in youth with bipolar disorder. Biological Psychiatry, 58(7), 540–548. 10.1016/j.biopsych.2005.07.019 16199011

[r11] Esterman, M., Reagan, A., Liu, G., Turner, C., & DeGutis, J. (2014). Reward reveals dissociable aspects of sustained attention. Journal of Experimental Psychology: General, 143(6), 2287–2295. 10.1037/xge0000019 25313950

[r12] Fitousi, D. (2020). Linking the ex-Gaussian parameters to cognitive stages: Insights from the linear ballistic accumulator (LBA) model. The Quantitative Methods for Psychology, 16(2), 91–106. 10.20982/tqmp.16.2.p091

[r13] Fleck, D. E., Eliassen, J. C., Durling, M., Lamy, M., Adler, C. M., DelBello, M. P., Shear, P. K., Cerullo, M. A., Lee, J.-H., & Strakowski, S. M. (2012). Functional MRI of sustained attention in bipolar mania. Molecular Psychiatry, 17(3), Article 3. 10.1038/mp.2010.108 PMC3037439 20975662

[r14] Fleck, D. E., Sax, K. W., & Strakowski, S. M. (2001). Reaction time measures of sustained attention differentiate bipolar disorder from schizophrenia. Schizophrenia Research, 52(3), 251–259. 10.1016/S0920-9964(01)00170-0 11705718

[r15] Fortenbaugh, F. C., Degutis, J., & Esterman, M. (2017). Recent theoretical, neural, and clinical advances in sustained attention research. Annals of the New York Academy of Sciences, 1396, 70–91. 10.1111/nyas.13318 28260249 PMC5522184

[r16] Fox, M. D., Snyder, A. Z., Vincent, J. L., Corbetta, M., Van Essen, D. C., & Raichle, M. E. (2005). The human brain is intrinsically organized into dynamic, anticorrelated functional networks. Proceedings of the National Academy of Sciences, 102(27), 9673–9678. 10.1073/pnas.0504136102 PMC1157105 15976020

[r17] Fransson, P. (2005). Spontaneous low-frequency BOLD signal fluctuations: An fMRI investigation of the resting-state default mode of brain function hypothesis. Human Brain Mapping, 26(1), 15–29. 10.1002/hbm.20113 15852468 PMC6871700

[r18] Gallagher, P., Gray, J. M., Watson, S., Young, A. H., & Ferrier, I. N. (2014). Neurocognitive functioning in bipolar depression: A component structure analysis. Psychological Medicine, 44(5), 961–974. 10.1017/S0033291713001487 23800475

[r19] Gallagher, P., Nilsson, J., Finkelmeyer, A., Goshawk, M., Macritchie, K. A., Lloyd, A. J., Thompson, J. M., Porter, R. J., Young, A. H., Ferrier, I. N., McAllister-Williams, R. H., & Watson, S. (2015). Neurocognitive intra-individual variability in mood disorders: Effects on attentional response time distributions. Psychological Medicine, 45(14), 2985–2997. 10.1017/S0033291715000926 26073667

[r20] Gallagher, P. (2020). Neuropsychology of bipolar disorder. In A. H. Young, & M. F. Juruena (Eds.), Bipolar disorder: From neuroscience to treatment. current topics in behavioral neurosciences, vol 48. Springer, Cham. 10.1007/7854_2020_148 32671598

[r21] Gazzellini, S., Dettori, M., Amadori, F., Paoli, B., Napolitano, A., Mancini, F., & Ottaviani, C. (2016). Association between attention and heart rate fluctuations in pathological worriers. Frontiers in Human Neuroscience, 10. https://www.frontiersin.org/articles/10.3389/fnhum.2016.00648 10.3389/fnhum.2016.00648 PMC5187380 28082881

[r22] Gazzellini, S., Napolitano, A., Bauleo, G., Bisozzi, E., Lispi, M. L., Ardu, E., Castelli, E., & Benso, F. (2017). Time–frequency analyses of reaction times and theta/beta EEG ratio in pediatric patients with traumatic brain injury: A preliminary study. Developmental Neurorehabilitation, 20(7), 393–407. 10.1080/17518423.2016.1216470 27629793

[r23] Geurts, H. M., Grasman, R. P. P. P., Verté, S., Oosterlaan, J., Roeyers, H., van Kammen, S. M., & Sergeant, J. A. (2008). Intra-individual variability in ADHD, autism spectrum disorders and Tourette’s syndrome. Neuropsychologia, 46(13), 3030–3041. 10.1016/j.neuropsychologia.2008.06.013 18619477

[r24] Grahek, I., Shenhav, A., Musslick, S., Krebs, R. M., & Koster, E. H. W. (2019). Motivation and cognitive control in depression. Neuroscience & Biobehavioral Reviews, 102, 371–381. 10.1016/j.neubiorev.2019.04.011 31047891 PMC6642074

[r25] Harmell, A. L., Mausbach, B. T., Moore, R. C., Depp, C. A., Jeste, D. V., & Palmer, B. W. (2014). Longitudinal study of sustained attention in outpatients with bipolar disorder. Journal of the International Neuropsychological Society, 20(2), 230–237. 10.1017/S1355617713001422 24468127 PMC6342447

[r26] Heathcote, A., Popiel, S. J., Mewhort, D. J., & Butler, B. E. (1991). Analysis of response time distributions: An example using the Stroop task. Psychological Bulletin, 109(2), 340–347. 10.1037/0033-2909.109.2.340

[r27] Helps, S., James, C., Debener, S., Karl, A., & Sonuga-Barke, E. J. S. (2008). Very low frequency EEG oscillations and the resting brain in young adults: A preliminary study of localisation, stability and association with symptoms of inattention. Journal of Neural Transmission, 115(2), 279–285. 10.1007/s00702-007-0825-2 17994187

[r28] Hultsch, D. F., MacDonald, S. W. S., & Dixon, R. A. (2002). Variability in reaction time performance of younger and older adults. Journals of Gerontology - Series B Psychological Sciences and Social Sciences, 57(2), 101–115. 10.1093/geronb/57.2.P101 11867658

[r29] Johnson, K. A., Kelly, S. P., Bellgrove, M. A., Barry, E., Cox, M., Gill, M., & Robertson, I. H. (2007). Response variability in attention deficit hyperactivity disorder: Evidence for neuropsychological heterogeneity. Neuropsychologia, 45(4), 630–638. 10.1016/j.neuropsychologia.2006.03.034 17157885

[r30] Karalunas, S. L., Geurts, H. M., Konrad, K., Bender, S., & Nigg, J. T. (2014). Annual research review: Reaction time variability in ADHD and autism spectrum disorders: measurement and mechanisms of a proposed trans-diagnostic phenotype. Journal of Child Psychology and Psychiatry, and Allied Disciplines, 55(6), 685–710. 10.1111/jcpp.12217 24628425 PMC4267725

[r31] Kim, D., Kim, J., Koo, T., Yun, H., & Won, S. (2015). Shared and distinct neurocognitive endophenotypes of schizophrenia and psychotic bipolar disorder. Clinical Psychopharmacology and Neuroscience, 13(1), 94–102. 10.9758/cpn.2015.13.1.94 25912542 PMC4423159

[r32] Koetsier, G. C., Volkers, A. C., Tulen, J. H. M., Passchier, J., van den Broek, W. W., & Bruijn, J. A. (2002). CPT performance in major depressive disorder before and after treatment with imipramine or fluvoxamine. Journal of Psychiatric Research, 36(6), 391–397. 10.1016/S0022-3956(02)00026-2 12393308

[r33] Kolur, U. S., Reddy, Y. C. J., John, J. P., Kandavel, T., & Jain, S. (2006). Sustained attention and executive functions in euthymic young people with bipolar disorder. The British Journal of Psychiatry, 189(5), 453–458. 10.1192/bjp.bp.106.022921 17077437

[r34] Little, B., Anwyll, M., Norsworthy, L., Corbett, L., Schultz-Froggatt, M., Gallagher, P. (2024). Processing speed and sustained attention in bipolar disorder and major depressive disorder: A systematic review and meta-analysis. Bipolar Disorders, 26(2):109–128. doi: 10.1111/bdi.13396. 37973384

[r35] Liu, S. K., Chiu, C.- H., Chang, C.-J., Hwang, T.-J., Hwu, H.-G., & Chen, W. J. (2002). Deficits in sustained attention in Schizophrenia and affective disorders: Stable versus state-dependent markers. American Journal of Psychiatry, 159(6), 975–982. 10.1176/appi.ajp.159.6.975 12042186

[r36] Luce, R. D. (1986). Response times: Their role in inferring elementary mental organization. Oxford University Press.

[r37] Machida, K., Barry, E., Mulligan, A., Gill, M., Robertson, I. H., Lewis, F. C., Green, B., Kelly, S. P., Bellgrove, M. A., & Johnson, K. A. (2022). Which measures from a sustained attention task best predict ADHD group membership? Journal of Attention Disorders, 26(11), 1471–1482. 10.1177/10870547221081266 35253511

[r38] Macritchie, K. A. N., Lloyd, A. J., Bastin, M. E., Vasudev, K., Gallagher, P., Eyre, R., Marshall, I., Wardlaw, J. M., Ferrier, I. N., Moore, P. B., & Young, A. H. (2010). White matter microstructural abnormalities in euthymic bipolar disorder. British Journal of Psychiatry, 196(1), 52–58. 10.1192/bjp.bp.108.058586 20044661

[r39] Marchetti, I., Koster, E. H. W., Sonuga-Barke, E. J., & De Raedt, R. (2012). The default mode network and recurrent depression: A neurobiological model of cognitive risk factors. Neuropsychology Review, 22(3), 229–251. 10.1007/s11065-012-9199-9 22569771

[r40] Marotta, A., Chiaie, R. D., Spagna, A., Bernabei, L., Sciarretta, M., Roca, J., Biondi, M., & Casagrande, M. (2015). Impaired conflict resolution and vigilance in euthymic bipolar disorder. Psychiatry Research, 229(1–2), 490–496. 10.1016/j.psychres.2015.06.026 26144587

[r41] Martino, M., Magioncalda, P., Huang, Z., Conio, B., Piaggio, N., Duncan, N. W., Rocchi, G., Escelsior, A., Marozzi, V., Wolff, A., Inglese, M., Amore, M., & Northoff, G. (2016). Contrasting variability patterns in the default mode and sensorimotor networks balance in bipolar depression and mania. Proceedings of the National Academy of Sciences of the United States of America, 113(17), 4824–4829. 10.1073/pnas.1517558113 27071087 PMC4855585

[r42] Massidda, D. (2013). *Retimes: Reaction time analysis* [Computer software]. https://cran.r-project.org/package=retimes

[r43] Monto, S., Palva, S., Voipio, J., & Palva, J. M. (2008). Very slow EEG fluctuations predict the dynamics of stimulus detection and oscillation amplitudes in humans. Journal of Neuroscience, 28(33), 8268–8272. 10.1523/JNEUROSCI.1910-08.2008 18701689 PMC6670577

[r44] Naim-Feil, J., Bradshaw, J. L., Sheppard, D. M., Rosenberg, O., Levkovitz, Y., Dannon, P., Fitzgerald, P. B., Isserles, M., & Zangen, A. (2015). Neuromodulation of attentional control in major depression: A pilot deepTMS study. Neural Plasticity, 2016, e5760141. 10.1155/2016/5760141 PMC4707329 26823985

[r45] Paelecke-Habermann, Y., Pohl, J., & Leplow, B. (2005). Attention and executive functions in remitted major depression patients. Journal of Affective Disorders, 89(1–3), 125–135. 10.1016/j.jad.2005.09.006 16324752

[r46] Parasuraman, R., & Davies, D. R. (1977). A taxonomic analysis of vigilance performance. In Vigilance (pp. 559–574). Springer US. 10.1007/978-1-4684-2529-1_26

[r47] Parris, B., Dienes, Z., & Hodgson, T. (2013). Application of the ex-Gaussian function to the effect of the word blindness suggestion on stroop task performance suggests no word blindness. Frontiers in Psychology, 4. https://www.frontiersin.org/articles/10.3389/fpsyg.2013.00647 10.3389/fpsyg.2013.00647 PMC3778318 24065947

[r48] Poletti, S., Bollettini, I., Mazza, E., Locatelli, C., Radaelli, D., Vai, B., Smeraldi, E., Colombo, C., & Benedetti, F. (2015). Cognitive performances associate with measures of white matter integrity in bipolar disorder. Journal of Affective Disorders, 174, 342–352. 10.1016/j.jad.2014.12.030 25553397

[r49] Porter, R. J., Gallagher, P., Thompson, J. M., & Young, A. H. (2003). Neurocognitive impairment in drug-free patients with major depressive disorder. British Journal of Psychiatry, 182(MAR.), 214–220. 10.1192/bjp.182.3.214 12611784

[r50] R Core Team. (2021). *R: A language and environment for statistical computing* [Computer software]. R Foundation for Statistical Computing, Vienna, Austria. https://www.r-project.org/

[r51] Raichle, M. E., MacLeod, A. M., Snyder, A. Z., Powers, W. J., Gusnard, D. A., & Shulman, G. L. (2001). A default mode of brain function. Proceedings of the National Academy of Sciences, 98(2), 676–682. 10.1073/pnas.98.2.676 PMC14647 11209064

[r52] Ramsay, J. O., & Silverman, B. W. (2005). Functional data analysis. 10.1007/B98888

[r53] Revelle, W. (2022). *Psych: Procedures for psychological, psychometric, and personality research* (Version 2.2.9) [Computer software]. https://CRAN.R-project.org/package=psych

[r54] Rights, J. D., & Sterba, S. K. (2019). Quantifying explained variance in multilevel models: An integrative framework for defining R-squared measures. Psychological Methods, 24(3), 309–338. 10.1037/met0000184 29999378

[r55] Robinson, L. J., Thompson, J. M., Gallagher, P., Gray, J. M., Young, A. H., & Ferrier, I. N. (2013). Performance monitoring and executive control of attention in euthymic bipolar disorder: Employing the CPT-AX paradigm. Psychiatry Research, 210(2), 457–464. 10.1016/j.psychres.2013.06.039 23880481

[r56] Rodríguez-Cano, E., Alonso-Lana, S., Sarró, S., Fernández-Corcuera, P., Goikolea, J. M., Vieta, E., Maristany, T., Salvador, R., McKenna, P. J., & Pomarol-Clotet, E. (2017). Differential failure to deactivate the default mode network in unipolar and bipolar depression. Bipolar Disorders, 19(5), 386–395. 10.1111/bdi.12517 28714580

[r57] Ruf, T. (1999). The Lomb-Scargle periodogram in biological rhythm research: Analysis of incomplete and unequally spaced time-series. Biological Rhythm Research, 30(2), 178–201. 10.1076/brhm.30.2.178.1422

[r58] Ruf, T., & Astropy, partially based on C. original by P. et al (Numerical R. and the P. module. (2022). *Lomb: Lomb-scargle periodogram* (Version 2.1.0) [Computer software]. https://CRAN.R-project.org/package=lomb

[r59] Schmidt, G. J., Barbosa, A. O., de Assis, S. G., Nicaretta, D. H., & Schmidt, S. L. (2021). Attentional subdomains’ deficits in Brazilian patients with major depressive episodes. Neuropsychology, 35(2), 232–240. 10.1037/neu0000719 33764113

[r60] Shalev, N., Bauer, A. K. R., & Nobre, A. C. (2019). The tempos of performance. Current Opinion in Psychology, 29, 254–260. 10.1016/j.copsyc.2019.06.003 31302478 PMC6996131

[r61] Smith, B. (2013). Depression and motivation. Phenomenology and the Cognitive Sciences, 12(4), 615–635. 10.1007/s11097-012-9264-0

[r62] Sonuga-Barke, E. J. S., & Castellanos, F. X. (2007). Spontaneous attentional fluctuations in impaired states and pathological conditions: A neurobiological hypothesis. Neuroscience and Biobehavioral Reviews, 31(7), 977–986. 10.1016/j.neubiorev.2007.02.005 17445893

[r63] Stanislaw, H., & Todorov, N. (1999). Calculating of signal detection theory measures. Behavior Research Methods, Instruments, & Computers, 31(1), 137–149. 10.3758/bf03207704 10495845

[r64] Tamnes, C. K., Fjell, A. M., Westlye, L. T., Østby, Y., & Walhovd, K. B. (2012). Becoming consistent: Developmental reductions in intraindividual variability in reaction time are related to white matter integrity. The Journal of Neuroscience: The Official Journal of the Society for Neuroscience, 32(3), 972–982. 10.1523/JNEUROSCI.4779-11.2012 22262895 PMC6621149

[r65] Thompson, J. M., Gallagher, P., Hughes, J. H., Watson, S., Gray, J. M., Ferrier, I. N., & Young, A. H. (2005). Neurocognitive impairment in euthymic patients with bipolar affective disorder. British Journal of Psychiatry, 186(JAN.), 32–40. 10.1192/bjp.186.1.32 15630121

[r66] Thomson, D. R., Besner, D., & Smilek, D. (2015). A resource-control account of sustained attention: Evidence from mind-wandering and vigilance paradigms. Perspectives on Psychological Science, 10(1), 82–96. 10.1177/1745691614556681 25910383

[r67] Van Der Meere, J., Börger, N., & Van Os, T. (2007). Sustained attention in major unipolar depression. Perceptual and Motor Skills, 104(3_suppl), 1350–1354. 10.2466/pms.104.4.1350-1354 17879669

[r68] VanRullen, R. (2018). Attention cycles. Neuron, 99(4), 632–634. 10.1016/j.neuron.2018.08.006 30138586

[r69] Vaurio, R. G., Simmonds, D. J., & Mostofsky, S. H. (2009). Increased intra-individual reaction time variability in attention-deficit/hyperactivity disorder across response inhibition tasks with different cognitive demands. Neuropsychologia, 47(12), 2389–2396. 10.1016/j.neuropsychologia.2009.01.022 19552927 PMC4766847

[r70] Warm, J. S., Parasuraman, R., & Matthews, G. (2008). Vigilance requires hard mental work and is stressful. Human Factors: The Journal of the Human Factors and Ergonomics Society, 50(3), 433–441. 10.1518/001872008X312152 18689050

[r71] Wickham, H., Averick, M., Bryan, J., Chang, W., D’, L., Mcgowan, A., François, R., Grolemund, G., Hayes, A., Henry, L., Hester, J., Kuhn, M., Lin Pedersen, T., Miller, E., Bache, S. M., Müller, K., Ooms, J., Robinson, D., Seidel, D. P., … Yutani, H. (2019). Welcome to the tidyverse. Journal of Open Source Software, 4(43), 1686. 10.21105/JOSS.01686

[r72] Zhang, H., Yang, S. Y., Qiao, Y., Ge, Q., Tang, Y. Y., Northoff, G., & Zang, Y. F. (2022). Default mode network mediates low-frequency fluctuations in brain activity and behavior during sustained attention. Human Brain Mapping, *July*, 1–12. 10.1002/hbm.26024 PMC9704793 35903957

